# Pre-analytical mysteries: A case of severe hypervitaminosis D and mild hypercalcaemia

**DOI:** 10.11613/BM.2021.011001

**Published:** 2020-12-15

**Authors:** Emma Whittle, Elzahn de Waal, Tony Huynh, Oliver Treacy, Adam Morton

**Affiliations:** 1 Department of Endocrinology and Diabetes, Mater Hospital, South Brisbane QLD, Australia; 2 Department of Chemical Pathology, Mater Pathology, South Brisbane QLD, Australia; 3 Department of Endocrinology and Diabetes, Queensland Children’s Hospital, South Brisbane QLD, Australia; 4 Faculty of Medicine, University of Queensland, Brisbane QLD, Australia

**Keywords:** endocrinology, hypercalcaemia, hypervitaminosis D, interferences, paraprotein

## Abstract

We describe a case of severe hypervitaminosis D and mild hypercalcaemia in a 68-year-old woman who presented with fatigue and weight loss. Her 25-hydroxy vitamin D (25OHD) was > 400 nmol/L (50-150) and corrected serum calcium was 2.83 mmol/L (2.1-2.6). Her intact parathyroid hormone (PTH) was 4.9 pmol/L (2.0-9.5). Further investigation revealed an IgM kappa paraprotein, and a bone marrow aspirate confirmed a diagnosis of lymphoplasmacytic lymphoma/Waldenstrom’s macroglobulinemia (LPL/WM). As the vitamin D level was discordant with the patient’s other results and presentation, the presence of an assay interferent was suspected. A 1-in-2 dilution of the sample returned a 25OHD result of 84 nmol/L in keeping with the presence of an interferent. Testing for rheumatoid factor was negative. The sample was treated with an antibody blocking reagent (Scantibodies) and results were not consistent with heterophile antibody interference. The sample was then analysed using liquid chromatography tandem mass spectrometry (LC-MS/MS), which returned a 25OHD result of 82 nmol/L. Testing on an alternative immunoassay platform produced a 25OHD result of 75 nmol/L. Reapeted testing on the original platform following reduction of the monoclonal paraprotein with chemotherapy, returned a result of 64 nmol/L. The patient’s mild hypercalcaemia persisted following resolution of the monoclonal paraprotein, in keeping with a diagnosis of primary hyperparathyroidism. This case highlights the potential for paraproteins to cause assay interference, and the importance of considering interference when results are incongruous with the clinical presentation.

## Introduction

Hypervitaminosis D is a rare condition. Possible causes of hypervitaminosis D include prolonged ingestion of large doses of cholecalciferol, deficiency or variants in CYP24A1, and an artefactual result due to assay interference.

Hypervitaminosis D due to excessive oral or intramuscular supplementation is rare. Development of hypercalcaemia due to excessive oral cholecalciferol requires prolonged ingestion of doses in the order of 40,000 IU daily for at least six months. A case series from India reported on 15 patients with hypercalcaemia secondary to intramuscular administration of cholecalciferol (*1*). The shortest period to develop hypercalcaemia was five weeks, during which time the patient received 3.000,000 IU of vitamin D. Hypervitaminosis D due to excessive exogenous intake is associated with severe hypercalcaemia, low PTH, and normal phosphate levels. CYP24A1 deficiency is a rare cause of an elevated 25-hydroxy vitamin D (25OHD) and hypercalcaemia. The CYP24A1 gene encodes vitamin D 24 hydroxylase which metabolises both 25OHD and 1,25-dihydroxyvitamin D (1,25(OH)_2_D) to inactive metabolites 24,25-dihydroxyvitamin D (24,25(OH)_2_D) and calcitroic acid (*2*). Expression of CYP24A1 is usually induced by both hypercalcaemia and 1,25(OH)_2_D, thereby preventing vitamin D-induced hypercalcaemia. CYP24A1 deficiency was first described in infants but can present at any age. CYP24A1 variants/deficiency are characterised biochemically by hypercalcaemia, hypercalciuria, undetectable PTH and parathyroid-hormone related peptide, low 24,25(OH)_2_ D_3_, and elevated 1,25(OH)_2_ D_3_. Clinical manifestations include nephrolithiasis and nephrocalcinosis. A definitive diagnosis can be made by genetic testing. Seven cases of hypercalcaemia with onset during pregnancy have been described in women with CYP24A1 variants/deficiency, in the setting of 25OHD 1-alpha-hydroxylase expression by the placenta and enzyme upregulation in the maternal kidney (*2*).

Assay interference is another possible cause of an elevated 25OHD, and several possible interferents have been described, including biotin, polymyxin E (colistin), and paraproteins. The most commonly used techniques to measure 25OHD are automated immunoassays which may be affected by interference.

We describe a case of severe hypervitaminosis D and mild hypercalcaemia which highlights the importance of considering assay interference, the rare nature of hypervitaminosis D and its causes, and the diagnostic approach to hypercalcaemia in the setting of a normal parathyroid hormone (PTH) level.

## Case report

A 68-year-old woman presented to her family physician with fatigue and 5 kg of weight loss over a six-month period. Her past medical history was significant for a hiatus hernia and hypercholesterolaemia. Her regular medications included rabeprazole and rosuvastatin. She intermittently took cholecalciferol 1000 IU/day. Physical examination was unremarkable except for pallor.

The following instrument platforms were used in the initial investigation of the case: serum chemistry, including total calcium, phosphate, albumin, creatinine, and globulins, on the Vitros (Ortho Clinical Diagnostics, Rochester NY, USA); ionized calcium on the Radiometer ABL800 (Radiometer Medical, Brønshøj, Denmark); PTH and 25OHD on the Abbott Architect (Abbott Laboratories, Chicago, Illinois, USA); 1,25(OH)_2_D on the Diasorin Liaison XL (DiaSorin, Saluggia, Italy); and serum electrophoresis (sELP) on the Sebia HYDRASYS 2 (Sebia, Paris, France).

**Figure 1:**
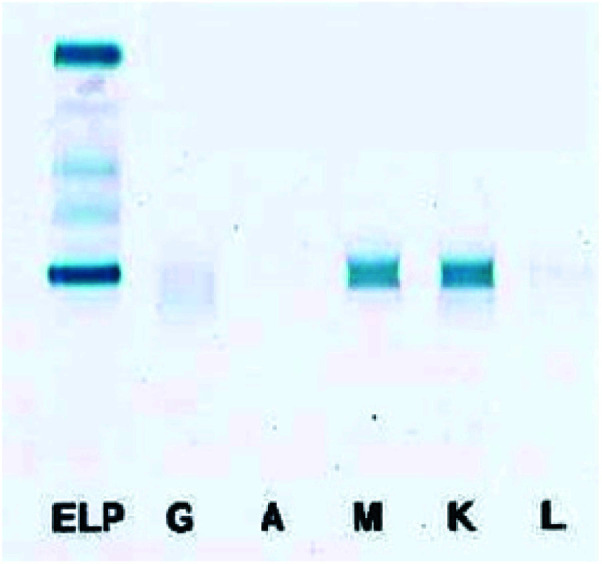
Electrophoresis gel showing a prominent IgM kappa paraprotein in the gamma region. ELP - serum protein electrophoresis. G - immunoglobulin G. A - immunoglobulin A. M - immunoglobulin M. K - kappa immunoglobulin; L - lambda immunoglobulin.

Initial investigations revealed haemoglobin of 87 g/L (115-160), corrected serum calcium of 2.83 mmol/L (2.1-2.6), serum albumin of 39 g/L (33-47), ionized calcium of 1.33mmol/L (1.13-1.3), and serum globulins of 61g/L (24-41). Further investigations in hospital showed an intact PTH of 4.9pmol/L (2.0-9.5), 25OHD of > 400nmol/L (50-150), 1,25(OH)_2_D of 152pmol/L (48-190), phosphate of 1.44 mmol/L (0.9-1.6), creatinine of 74 µmol/L (45-90), and an IgM kappa paraprotein in the gamma region of 44 g/L on sELP (Figure 1). The PTH result was confirmed using the Beckman Coulter DxI 800 (Brea, California, United States).

A positron emission tomography/computer tomography scan showed extensive mildly fluorodeoxyglucose (FDG) avid lymphadenopathy above and below the diaphragm, with large FDG avid mass lesions in the pelvis, and widespread bone marrow infiltration. A bone marrow aspirate showed moderate to heavy marrow involvement with a mature lymphoproliferative disorder, consistent with a diagnosis of lymphoplasmacytic lymphoma/Waldenstrom’s macroglobulinaemia (LPL/WM).

The 25OHD level was felt to be inconsistent with clinical findings and the possibility of an aberrant result was considered.

### Laboratory analyses

**Figure 2:**
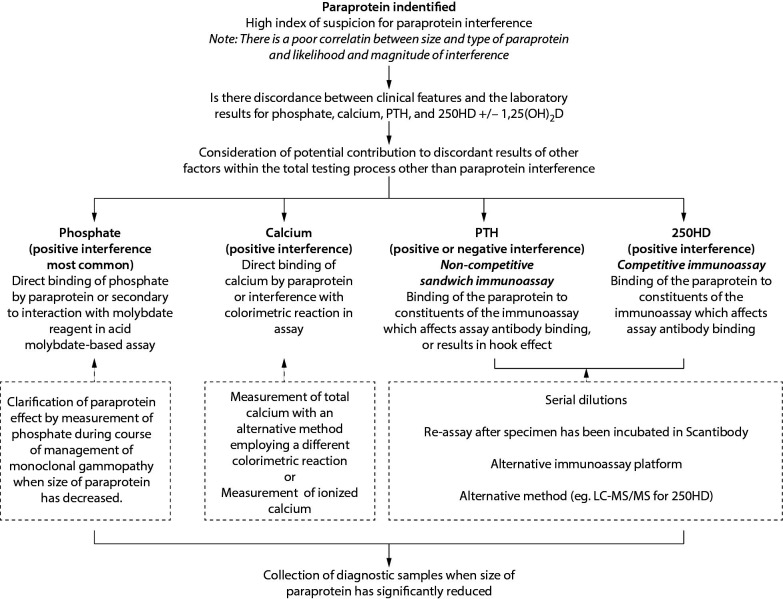
Suggested algorithm for the investigation of potential paraprotein interference in disorders of calcium and phosphate homeostasis.

Vitamin D results using different assays are summarised in table 1. The steps taken to confirm interference are summarised in figure 2. The 25OHD result of > 400 nmol/L was confirmed on repeat analysis using the Abbott Architect immunoassay platform. In order to confirm our suspicions, a series of steps was taken. Firstly, a 1-in-2 dilution of the sample with Architect Multi-Assay Manual Diluent returned a result of 84 nmol/L – highly suggestive of the presence of an interferent. The presence of rheumatoid factor was excluded with a result of < 15 IU/L (reference range < 30 IU/L, Abbott Architect, Abbott Laboratories, Chicago, Illinois, USA). Treatment of the sample with antibody blocking reagent (Scantibodies, Santee, California, United States) was not consistent with heterophile antibody interference. Analysis of 25OHD *via* liquid chromatography tandem mass spectrometry (LC-MS/MS) returned a result of 82 nmol/L (Waters LC-MS/MS, Milford, Massachusetts, United States). Measurement of the sample on an alternative immunoassay platform (Siemens Centaur, Bellport, New York, United States), produced a result of 75 nmol/L, in agreement with the LC-MS/MS result. Following chemotherapy, and in the setting of normal globulins, the patient’s repeat 25OHD result on the Abbott Architect instrument was 64 nmol/L, consistent with the monoclonal paraprotein interfering with the original assay.

**Table 1:** Summary of 25OHD results

	**Abbott ARCHITECT** **(Neat)**	**Abbott ARCHITECT** **(Scantibodies** **Treated)**	**Abbott ARCHITECT** **(1-in-2 dilution)**	**Siemens** **CENTAUR** **(Neat)**	**LC-MS/MS** **(Neat)**	**Abbott ARCHITECT** **After 3 weeks of chemotherapy** **(Neat)**
**Vitamin D, nmol/L**	> 400	> 400	84	75	82	64
LC-MS/MS – liquid chromatography tandem mass spectrometry						

At the time of the patient’s initial presentation, a 24-hour urine collection demonstrated calcium excretion of 1.8 mmol/day, with a calcium:creatinine clearance ratio (CCCR) of 0.005. On questioning previous pathology providers, multiple corrected serum calcium levels between 2011 and 2016 had been normal. No relatives were available for screening for hypercalcaemia. Bone densitometry was assessed following her discharge from hospital and was found to be normal.

## Discussion

The most striking feature of this patient’s initial investigations was the 25OHD level of > 400nmol/L. Based on the patient’s history, hypervitaminosis D secondary to excess supplementation or CYP24A1 deficiency were thought to be unlikely. 25OHD assay interference was identified as the most likely explanation.

25-hydroxy vitamin D is typically measured using automated immunoassays. The vitamin D External Quality Assessment Scheme (DEQAS) includes 26 different methods or variants of methods for measurement of 25OHD (*3*). The average coefficient of variation with immunoassays is approximately 10-15%, however may be as high as 20-30% (*4*). LC-MS/MS methods are more consistent (coefficient of variation < 5-10%),more accurate (bias < 5%), and have the advantage of being able to accurately quantitate both 25OHD_2_ and 25OHD_3_ when compared to antibody-based methods. LC-MS/MS instruments are more expensive and have a lower sample throughput (*5*). Other vitamin D metabolites may interfere with 25OHD assays resulting in overestimation of total 25OHD. The 3-epi-25OHD_3_ may interfere with LC-MS/MS and high performance liquid chromatography assays, and 24R,25(OH)_2_D_3_, and 24S,25(OH)_2_D_3_ may interfere in immunochemical and protein binding assays (*3*, *6*, *7*). Biotin ingestion may result in significant overestimation of 25OHD by some assays (*8*, *9*). A case report described significant interference by polymyxin E (colistin) therapy with multiple assays including 25OHD in a critically ill patient (*10*). We were also unable to find any reports of heterophile antibodies interfering with 25OHD assays.

Artefactually elevated 25OHD levels on chemiluminescent immunoassays have been reported in two patients with myeloma-related monoclonal immunoglobulin peaks (*11*, *12*). One of these patients also had long-standing rheumatoid arthritis, though we were unable to find any other cases of interference with 25OHD assays by rheumatoid factor. Once a paraprotein has been identified, clinicians should maintain a high index of suspicion for potential interference in laboratory assays. There is a poor correlation between paraprotein size and type, and the likelihood and magnitude of the paraprotein interference (*13*). Results of investigations for suspected disorders of calcium and phosphate homeostasis which are not consistent with the clinical features or exhibit discordance should prompt further evaluation. The clinical laboratory should be consulted early in the evaluation process to provide details regarding specific laboratory methods, facilitate interference testing, and to organise the analysis of samples in other laboratories employing alternative immunoassay platforms and alternative methods.

Investigation of erroneous results should include interrogation of the total testing process, from ensuring the correct test was ordered to the correct interpretation of test results (*14*). Simple paraprotein-related effects, such as failed aspiration of sample on the automated chemistry analyser due to hyperviscosity, should be excluded. Directed strategies to exclude paraprotein interference depend on the measurand of interest. Paraprotein interference with routine phosphate quantitation results either from direct paraprotein binding of phosphate, or secondary to interaction with the universal routine acid molybdate-based methods. Positive interference is more commonly observed, but negative interference has been described (*15*). Interference testing for phosphate is often limited to re-measurement of phosphate following significant reduction in the size of the paraprotein. The measurement of total calcium is achieved *via* assay-specific colorimetric reactions. Paraprotein interference can be investigated by using an alternative platform which utilises a different colorimetric reaction, or by measurement of ionized calcium. PTH and 25OHD are routinely measured by immunoassays. Paraprotein interference in immunoassays results from binding of the paraprotein to constituents of the immunoassay which affects antibody binding (*16*). For non-competitive sandwich immunoassays, such as PTH assays, paraproteins can theoretically cause either positive or negative interference as well as the hook effect in one-step assays. There have been no published cases of paraprotein interference in PTH measurement. Immunoassay-based 25OHD methods are competitive assays. Positive interference from paraproteins is well-recognized. Standard interference testing strategies such as assessment of linearity with serial dilutions, re-analysis following Scantibody treatment, and analysis with an alternative immunoassay platform should be considered. For 25OHD, the relative wide accessibility of LC-MS/MS-based methods provides a robust approach to exclude a potential paraprotein effect. Clinicians should consider re-analysis of diagnostic samples, which may necessitate with holding therapies for provisionally diagnosed disorders of calcium and phosphate homeostasis, following adequate management of the monoclonal gammopathy.

In our patient, the presence of hypercalcaemia and a non-suppressed PTH further complicated the clinical picture. Hypercalcaemia with a non-suppressed PTH may occur with primary hyperparathyroidism (PHPT), familial hypocalciuric hypercalcaemia (FHH) and pseudohypercalcaemia (*17*). Factitious or pseudohypercalcaemia may occur due to hyperalbuminaemia, thrombocytosis, or assay interference by paraproteins. Pseudohypercalcaemia leading to the incorrect diagnosis of PHPT has been reported with LPL/WM, multiple myeloma, monoclonal gammopathy of uncertain significance, mixed cryoglobulinaemia with Sjögren’s syndrome, and markedly elevated IgE levels. Pseudohypercalcaemia may be identified by the demonstration of normal ionised calcium levels. In the case presented, the patient’s ionised calcium was mildly elevated at 1.33 mmol/L (1.13-1.30), excluding pseudohypercalcaemia.

Differentiating FHH from asymptomatic PHPT may be difficult because of considerable overlap in CCCR, serum calcium, serum phosphate, serum magnesium, and PTH between the two disorders (*18*). PHPT is much more common than FHH. The prevalence of FHH in the west of Scotland was estimated to be 1 in 78,000 compared with the estimated incidence of PHPT of 50 cases *per* 100,000 patient years (*19*, *20*). While PHPT is usually characterised by an elevated PTH level, 10 to 20% of patients will have an inappropriately “normal” PTH level. The CCCR is the consensus biochemical measurement to try to distinguish PHPT from FHH (*21*, *22*). The 2009 consensus panel on Guidelines in the Management of Asymptomatic PHPT set the CCCR threshold value at less than 0.01 for the diagnosis of FHH, and greater than 0.02 for the diagnosis of PHPT (*23*). However there is considerable overlap in CCCR in the two conditions, with up to 20% of individuals with FHH having a CCCR > 0.01, and up to 18.2% of individuals with surgically proven PHPT having preoperative CCCR < 0.01 (*24*, *25*). CCCR may be lowered with vitamin D deficiency, renal dysfunction, old age, low dietary calcium intake, and the use of thiazide diuretics and anti-resorptive agents for osteoporosis. Pregnancy leads to an elevated CCCR in the setting of physiological hypercalciuria, making differentiation of FHH and PHPT difficult (*26*). FHH is an inherited autosomal dominant condition with almost complete penetrance. The majority of mutations are linked to the gene encoding the calcium-sensing receptor on the long arm of chromosome 3 (*19*). New mutations are relatively rare. In cases where biochemical differentiation between PHPT and FHH is uncertain, testing of first-degree relatives or genetic testing may be useful.

While the patient’s CCCR is low, the previous normal corrected serum calcium results make it most likely the patient has PHPT. In view of the patient’s normal bone density, mild degree of hypercalcaemia, the absence of relatives for testing, and her diagnosis of LPL/WM, it was decided not to pursue gene testing to differentiate between the two disorders as it would not change management. On review of the literature, there does not appear to be an association between WM/LPL and PHPT.

In conclusion, this case highlights the potential for immunoassay interference when measuring 25OHD, and the potential for paraproteins to cause interference. The patient’s history and presentation were not in keeping with vitamin D intoxication, which prompted consideration of other causes of an elevated 25OHD level. The presence of an elevated ionised calcium confirmed hypercalcaemia, as pseudohypercalcaemia should be considered in patients with WM. The most likely cause of hypercalcaemia in this patient is PHPT, a condition which is not typically associated with WM/LPL.

## What can be done to prevent such errors?

Close liaison between physicians and clinical biochemists is essential in the setting of laboratory results that are unexpected and/or incongruous with the patient’s clinical picture to exclude assay interference.
